# Credibility, Accuracy, and Comprehensiveness of Readily Available Internet-Based Information on Treatment and Management of Peripheral Artery Disease and Intermittent Claudication: Review

**DOI:** 10.2196/39555

**Published:** 2022-10-17

**Authors:** Shelley Alexander, Chris Seenan

**Affiliations:** 1 Department of Physiotherapy and Paramedicine School of Health and Life Sciences Glasgow Caledonian University Glasgow United Kingdom

**Keywords:** peripheral artery disease, intermittent claudication, health information, education, internet, eHealth, digital health

## Abstract

**Background:**

Peripheral artery disease (PAD) affects millions of people worldwide, and a core component of management of the condition is self-management. The internet is an important source of health information for many people. However, the content of websites regarding treatment recommendations for PAD has not been fully evaluated.

**Objective:**

This study aimed to assess the credibility, accuracy, and comprehensiveness of websites found via a common search engine, by comparing the content to current guidelines for treatment and management of PAD and intermittent claudication (IC).

**Methods:**

A review of websites from hospitals, universities, governments, consumer organizations, and professional associations in the United States and the United Kingdom was conducted. Website recommendations for the treatment of PAD and IC were coded in accordance with the guidelines of the National Institute for Health and Care Excellence (NICE) and the American Heart Association (AHA). Primary outcomes were website credibility (4-item Journal of the American Medical Association benchmark), website accuracy (in terms of the percentage of accurate recommendations), and comprehensiveness of website recommendations (in terms of the percentage of guideline recommendations that were appropriately covered). Secondary outcomes were readability (Flesch–Kincaid grade level) and website quality (Health On the Net Foundation’s code of conduct).

**Results:**

After screening, 62 websites were included in this analysis. Only 45% (28/62) of websites met the credibility requirement by stating they were updated after the NICE guidelines were published. Declaration of authorship and funding and the presence of reference lists were less commonly reported. Regarding accuracy, 81% (556/685) of website recommendations were deemed accurate on following NICE’s and the AHA’s recommendations. Comprehensiveness was low, with an average of 40% (25/62) of guideline treatment recommendations being appropriately covered by websites. In most cases, readability scores revealed that the websites were too complex for web-based consumer health information.

**Conclusions:**

Web-based information from reputable sources about the treatment and management of PAD and IC are generally accurate but have low comprehensiveness, credibility, and readability.

## Introduction

The internet is increasingly being used by the general public as a source of health information [1]. People may use an internet search at various times along a health care journey: prior to seeking medical advice from their health care providers, to support self-management, and to make treatment decisions [1]. This is especially relevant to people with peripheral arterial disease (PAD), where self-management and behavior change are key aspects of care [2]. Clinical guidelines that summarize the best available research evidence and expert consensus for the diagnosis and management of PAD have been developed by the American Heart Association (AHA) [3] and the National Institute of Health and Care Excellence (NICE) [4]. These guidelines recommend lifestyle modifications including cessation of smoking, a healthy diet, sustaining a healthy weight, and regular physical activity. Prescriptions of antiplatelets, statins, antihypertensives, and vasodilators are also recommended, but stenting and bypass surgery should only be considered if structured exercise and lifestyle modifications have been exhausted [3,4].

Most commonly, search engines, such as Google, are used as the method of searching for health information on the internet [5]. A 2014 report found that 60% of UK respondents had used the internet to search for health information in the previous 12 months, with younger people being more likely to search for information in this manner than older generations [1]. It is likely for these figures to have increased, especially considering the global COVID-19 pandemic. A recent survey found that health services had been completely or partially disrupted in many countries as a result of the pandemic, including services for hypertension, diabetes, cancer, and cardiovascular emergencies [6]. This indicates that potentially less health care provision for people with PAD may have been available since the onset of the pandemic. Along with reductions in access to in-person health care, the number of people searching the internet for health care–related information, such as that on PAD, could have increased. It is also likely for older generations to use the internet more than they did previously, and as this is the demographic more likely to experience PAD, there may be more people searching for such information on the internet than ever before.

Owing to the importance of self-management in long-term conditions and the expanding role of the internet in gathering health information, it is essential that web-based sources of information are accurate, credible, and comprehensive [7]. However, despite the large number of people seeking web-based health information, previous research has found the quality of these websites to be relatively poor [8-11]. To date, only one study has assessed the quality of information about PAD and intermittent claudication (IC) on websites and videos [12], and no previous research has compared web-based information to clinical guidelines. By assessing the quality of this information and any gaps or inaccuracies within it, recommendations can be made to improve the quality of information on the internet while optimizing the quality of life for those living with PAD. Through accurate self-management advice and support with behavior change, internet searching could empower individuals to assume a more active role in managing their condition [10].

This review aims to compare trustworthy websites to current clinical guidelines for the treatment and management of PAD and IC to assess their credibility, accuracy, comprehensiveness, and readability.

## Methods

### Study Design

This review is reported in accordance with the Preferred Reporting Items for Systematic Reviews and Meta-Analyses guidelines where possible [13]. A review of “trustworthy” websites from governments, hospitals, universities, professional bodies, and health care organizations was conducted.

### Eligibility Criteria

Websites were sought from the United States and the United Kingdom and had to be written in English to be included. Websites were deemed trustworthy if they were from the government, nonprofit organizations, hospitals, universities, professional societies, or consumer organizations. To be included, websites had to mention at least one recommendation for the treatment or management of PAD or IC. Web links from Google searches, which directed us to PDFs, were included if they met every other criterion mentioned above. Websites were excluded if they were not freely accessible, required sign-in details, or required payment to be accessed. Any Google Ads on the 2 pages screened were excluded. Web links to other parts of the same website were followed and included, but web links leading to external sources were excluded. Websites were excluded if they were scientific journal articles, blogs, videos, or the comparative guidelines themselves.

### Search Strategy

A recent review has indicated that although people seeking health information on the web tend to use health websites, they often start with generic search engines, most commonly Google [5]. Google was used to search for freely accessible, noncommercial websites presenting information on PAD or IC during October 21-23, 2020, and updated during October 1-3, 2021. Search terms were decided after some trial searching on Google Trends. It was found that abbreviations “PAD” and “IC” were often searched for reasons other than those for “peripheral artery disease” and “intermittent claudication,” respectively; hence, the nonabbreviated versions were used as search terms. To target more trustworthy websites including government, hospital, university, and consumer organization websites, various words were added after the initial search terms [10]. A full breakdown of search terms is shown in Table 1. The search engine, Google, was used as it is considered the most common search engine with the best search validity [14]. For increased search specificity, each term was searched on both the UK (google.co.uk) and US (google.com) domains of Google. The first 2 pages of results were screened from each search in line with the eligibility criteria. The browsing data were cleared between each search. All web links deemed relevant by the first reviewer (SA) were collated to an Excel (Microsoft Inc) spreadsheet and then screened for eligibility by the second reviewer (CS), with all discrepancies resolved through discussion.

**Table 1:** Search terms used on Google; adapted from Ferreira et al [10].

Peripheral artery disease		Intermittent claudication
**United Kingdom (google.co.uk)**		
	*Peripheral Artery Disease gov uk*	*Intermittent Claudication gov uk*
	*Peripheral Artery Disease org uk*	*Intermittent Claudication org uk*
	*Peripheral Artery Disease hospital uk*	*Intermittent Claudication hospital uk*
	*peripheral Artery Disease university uk*	*Intermittent Claudication university uk*
	*Peripheral Artery Disease association society uk*	*Intermittent Claudication association society uk*
	*Peripheral Artery Disease consumer reports uk*	*Intermittent Claudication consumer reports uk*
**United States (google.com)**		
	*Peripheral Artery Disease gov usa*	*Intermittent Claudication gov usa*
	*Peripheral Artery Disease org usa*	*Intermittent Claudication org usa*
	*Peripheral Artery Disease hospital usa*	*Intermittent Claudication hospital usa*
	*peripheral Artery Disease university usa*	*Intermittent Claudication university usa*
	*Peripheral Artery Disease association society usa*	*Intermittent Claudication association society usa*
	*Peripheral Artery Disease consumer usa*	*Intermittent Claudication consumer usa*

### Data Extraction

Both reviewers (SA and CS) extracted data into separate spreadsheets then met to discuss and cross-check the data. Recommendations for PAD and IC treatment or management from each website were coded in accordance with the 2012 NICE recommendations (last updated in 2018) and the 2016 AHA guidelines for PAD and IC management and treatment [3,4]. There were minimal recommendations mentioned in one guideline but not in the other, and there were no conflicting recommendations. Each website recommendation was coded against the guidelines as endorsed by at least one guideline or dismissed by at least one guideline [10]. Treatment and management recommendations from websites were each compared to the combined guideline recommendations and coded, as seen in Table 2.

**Table 2:** Code for which websites were compared and graded [10].

Coding criteria	Description
Appropriate endorsement	A website recommendation to use a treatment that was also endorsed by at least 1 guideline.
Appropriate dismissal	A website recommendation to avoid a treatment that was also dismissed by at least 1 guideline.
Inappropriate endorsement	A website recommendation to use a treatment that was dismissed by at least 1 guideline.
Inappropriate dismissal	A website recommendation to avoid a treatment that was endorsed by at least 1 guideline.
Endorsed	A website recommendation to use a treatment not mentioned in either guideline.
Dismissed	A website recommendation to avoid a treatment not mentioned in either guideline.
Unclear	A website recommendation that was too vague to be clearly matched to the guidelines or led to discrepancies between researchers.

### Outcomes

#### Credibility

The credibility of each website was assessed using the Journal of the American Medical Association (JAMA) benchmark [10,15]. The JAMA benchmark evaluates websites on 4 items: (1) information currency, (2) authorship declaration, (3) presence of a reference list, and (4) disclosure of any conflicts of interest, sponsorship, or funding. Information was deemed current if it was dated after NICE guidelines were published (August 8, 2012) [16]. A declaration of authorship was included if single or multiple authors were mentioned, or authorship was tied to a group or entity [10]. Each of the 4 items was answered with “Yes,” “No,” or “Not reported.”

#### Accuracy

The number of recommendations from websites that were accurate and clear were defined as those that were coded as appropriate endorsements, appropriate dismissals, or dismissed treatments not mentioned in either guideline. Recommendations were deemed inaccurate if they were coded as inappropriate endorsements, inappropriate dismissals, or endorsed treatments not mentioned in either guideline [10].

#### Comprehensiveness

The proportion of accurate guideline recommendations covered by a website was determined to measure their comprehensiveness. Website comprehensiveness was determined from the ratio of the sum of appropriate endorsements and dismissals against the total number of recommendations in the comparative guidelines [10].

### Readability

The Flesch–Kincaid grade level (FKGL) [17] is widely accepted as an appropriate instrument to evaluate the readability of general and health documents. The FKGL yields a reading level score calculated from the average length of words and sentences in a discourse. The score yielded by the FKGL is rated against US school levels, and it has been suggested that the FKGL should be between 6 and 8 for medical and health information aimed at the general public [18,19]. In this study, the websites were segregated into 3 groups based on the FKGL: <8, 8-10, and >10. Websites with an FKGL of <8 are deemed accessible to most people, those scoring 8-10 are accessible to some, and those scoring >10 are deemed inaccessible to the majority of the UK or US readers. The FKGL was calculated in this study using the inbuilt readability function in Word (version 2013; Microsoft Inc). A large section of text was copied from each website and pasted onto Word before running the readability statistic.

### HONcode

The Health On the Net (HON) Foundation’s code of conduct (HONcode) is a well-known ethical and trustworthy code for evaluating the quality of medical and health information available on the internet. Websites that follow the HONcode principles can be approved by the HON foundation and be allowed to display the HONcode certificate symbol at the bottom of the page as a benchmark of quality. The HONcode has been used in similar previous studies as an indication of web-based health information quality [11,17,18]. In this review, the item was scored as “Yes” or “No” for presence of the HONcode logo on each website.

## Results

### Website Selection

**Figure 1:**
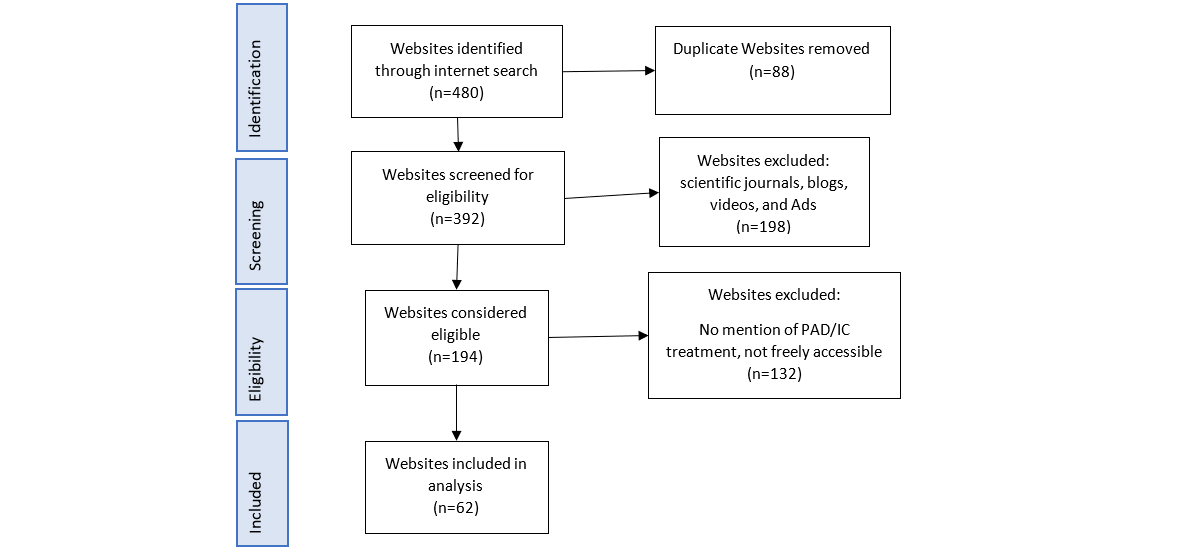
Study flow diagram with reasons for website exclusion. IC: intermittent claudication; PAD: peripheral artery disease.

Searches were conducted 24 individual times with the first 2 pages of results being assessed for eligibility. Searches collated 480 website results, and after duplicate results (n=88) were removed, the rest were screened for eligibility. Of these websites, 330 were deemed ineligible with reasons given in Figure 1. The remaining 62 websites were included in the analysis.

### Website Characteristics

From the UK- and US-specific Google sites searched, 48% (30/62) of eligible websites were found from the United Kingdom and 52% (32/62) of them from the United States. A large proportion of the analyzed websites (45%, n=28) were those of hospitals, followed by those of nongovernment organizations (23%, n=14), universities (13%, n=8), government organizations (10%, n=6), consumer organizations (5%, n=3) and, finally, professional associations or societies (5%, n=3). Information about the characteristics of the websites is provided in Table 3.

**Table 3:** Characteristics of websites and credibility data (N=62).

Descriptive and credibility variables		Websites, n (%)
**Country**		
	United Kingdom	30 (48)
	United States	32 (52)
**Website type**		
	Government	6 (10)
	Hospital	28 (45)
	University	8 (13)
	Consumer organization	3 (5)
	Nongovernment organization	14 (22)
	Professional association or society	3 (5)
**Updated in accordance with NICE^a^ guidelines (August 8, 2012)**		
	Yes	27 (44)
	No	2 (3)
	Not reported	33 (53)
**Authorship declared**		
	Yes	17 (27)
	No	45 (73)
**Contains a reference list**		
	Yes	15 (24)
	No	47 (76)
**Disclosure of conflicts of interest or funding**		
	Yes	1 (2)
	Not reported	61 (98)

^a^NICE: National Institute for Health and Care Excellence.

### Credibility

The date of publication or last review was present on 32 websites with 5 (8%) of these dated before the NICE guidelines were published and the other 27 (44%) dated after and therefore deemed as being up to date. However, 30 (48%) websites did not report a date on their web page. Authorship was declared on only 18 (29%) websites, and 15 (24%) websites presented a reference list. Disclosure of any conflicts of interest, sponsorship, or funding was declared on 1 (2%) website (Table 3). More details on the assessment of website credibility can be found in Multimedia Appendices 1-3.

### Accuracy

From the 62 websites analyzed, a total of 685 recommendations were recorded, with 556 (81.2%) being accurate, 10 (1.5%) inaccurate, and 114 (16.6%) unclear (Table 4). Most recommendations by websites were to use a treatment (n=589, 85.9%) rather than to avoid a treatment. The proportion of accurate recommendations was the highest from among UK searches (87.8%) in comparison to US searches (75.3%). Searches for IC yielded a higher proportion of accurate recommendations (86.9%) than searches for PAD (78.5%).

Further information on website recommendation accuracy is presented in Multimedia Appendices 1-3. The treatments most appropriately endorsed by websites were smoking cessation and cholesterol management (53/62, 86%), followed closely by angioplasty (n=52, 84%), physical activity (n=51, 82%), and blood pressure management (n=51, 82%). Least appropriately endorsed treatments included annual flu vaccine (0%) and exercise to maximal pain (n=6, 10%). Pentoxifylline was the most common treatment to be inappropriately endorsed by 5 (8%) websites, followed by anticoagulants (n=2, 3%). The most unclear recommendation was stenting, with 32 (52%) websites mentioning stenting but not in enough detail to match the comparative guidelines.

Importantly, none of the recommended treatments were inappropriately dismissed by any website. The most common website recommendation that was not mentioned in the guidelines was looking after mental well-being, which was mentioned in 6 (10%) websites. Website recommendations to avoid treatments not mentioned in the guidelines included the following: avoiding cold temperatures, not wearing compression stockings, and avoiding medication or herbal remedies that have been deemed ineffective or dangerous.

**Table 4:** Accuracy of website recommendations for the treatment of peripheral artery disease or intermittent claudication.

Search terms	Recommendations, n	Unclear recommendations, n	Accurate recommendations, n	Accurate endorsements, n	Accurate dismissals, n
*Peripheral Artery Disease UK*	208	31	178	173	2
*Peripheral Artery Disease USA*	262	49	191	187	0
*Intermittent Claudication UK*	113	12	104	99	2
*Intermittent Claudication USA*	102	22	83	75	4
Total	685	114	556	534	8

### Comprehensiveness

Overall comprehensiveness of the included websites was low, covering 38% of recommended guidelines, on average, with approximately 8 out of 21 accurate recommendations (Table 5). The most comprehensive website had 13 recommendations that clearly and accurately matched the comparative guidelines, resulting in a comprehensiveness of 62%. Ranging from 2 to 13 accurate recommendations, the comprehensiveness of the websites found was extremely varied (10%-62%). No website mentioned all recommended treatments from the guidelines and most mentioned less than half. Full details on comprehensiveness are provided in Multimedia Appendices 1-3.

**Table 5:** Comprehensiveness of recommendations for peripheral artery disease or intermittent claudication treatment by websites when compared to the guidelines of the National Institute for Health and Care Excellence and the American Heart Association.

	Guideline recommendations, n	Guideline recommendations accurately covered by websites, mean (SD; %^a^)
Recommendations to use a treatment	16	8.7 (3.1; 41.4)
Recommendations to avoid a treatment	5	0.2 (0.6; 0.9)
Total treatment recommendations	21	8.9 (3.7; 42.3)

^a^Percentage of total guideline recommendations.

### HONcode and Readability

Only 5 of 62 (8%) websites were found to have the HONcode logo displayed on their web page as a marker of website quality. Of the 62 websites, 3 (5%) had an FKGL of <8 as recommended for health information aimed at the general public [18]. An additional 17 (27%) websites had an FKGL of 8-10, and 42 (68%) websites scored >10. The FKGL for most of the websites (68%) is deemed too high, which would make it difficult for most of the population to comprehend the presented information (Table 6). The FKGL scores ranged from 5.7 to 16.4, which covers a vast range of reading levels.

**Table 6:** Website quality and readability results.

Evaluation instrument			Websites, n (%)
**Health On the Net Foundation’s code of conduct**			
	Yes	5 (8.1)	
	No	57 (91.9)	
**Flesch–Kincaid grade level**			
	<8	3 (4.8)	
	8-10	17 (27.4)	
	>10	42 (67.7)	

## Discussion

### Principal Findings

This is the first study to compare web-based information from trustworthy sources for people with PAD to current clinical guidelines to assess their credibility, accuracy, and comprehensiveness. Website recommendations for the treatment or management of PAD and IC were found to have low credibility when measured against the JAMA benchmark. Most recommendations provided were accurate; however, most websites lacked comprehensiveness and were not always clear in their recommendations. A high proportion of websites were too difficult for the average person to read and thus understand the recommendations they provided.

As this is the first study to assess web-based information regarding PAD and IC with respect to NICE and AHA guidelines, no direct comparisons to previous literature can be made. However, the 81.2% of accurate recommendations by websites found in this study is higher than that reported in a similar study on low back pain and pancreatic cancer, where only 43.3% and 55% of website treatment recommendations, respectively, were accurate [9,10]. Both of these studies included more results from their searches—the first 50 or 100 results from each search—than this study, which only included the first 20 results [10]. Screening more results may yield websites that are less related to the search terms on the latter pages and yield less accurate recommendations as a result. Individuals rarely look past the first 2 pages of search engine results; hence, screening the first 20 results (2 pages) will have covered the sites that people with PAD are most likely to view.

Even though the recommendation accuracy was high, comprehensiveness was low with websites, averaging 8 out of 21 accurate recommendations (38%) from the guidelines. This indicates that generally, websites do not go into enough depth about the variety of treatment options for PAD and IC. This finding is similar to that of a previous study, where websites covered 6.73 of 17 recommendations (40%) for low back pain on average [10].

Smoking is one of the strongest risk factors for PAD [20], and cessation in people with IC has been shown to reduce mortality [21]. This is reflected in the web-based information as smoking cessation was accurately recommended by 86% of websites. Lifestyle modifications are the first line of treatment for PAD and can reduce cardiovascular ischemic events and improve function [3]. Therefore, it is surprising that the next most appropriately endorsed recommendation from websites was angioplasty (n=52/62, 84%). Surgical procedures are not a first line of treatment for most people with PAD, but among the websites reviewed in this study, they are more commonly recommended than, for example, exercise. A large proportion of the analyzed websites were those of US hospitals (24/62, 39%), and these sites may be advocating more for the surgical services they provide. A study of web-based information on pancreatic cancer yielded similar findings, indicating that website recommendations from US treatment centers were focused on treatment options offered at their facilities [9]. While information provided on these websites is mostly accurate, it is not comprehensive enough and could introduce surgical bias, thus undermining the potential success of other management strategies.

The general lack of self-management information found on websites in this study is reflective of the overall attitudes toward PAD and IC treatment in both the United Kingdom and the United States. A recent review of patient experiences of PAD [22] reported that patients often have very limited understanding of their condition. Being unaware of the systemic nature of PAD while also lacking information on self-management techniques from health care professionals leads patients to believe that surgical interventions alone will “cure” them [23,24]. People with PAD are often not involved in treatment-related decision-making and believe that doctors and surgeons know best, leading to unrealistic expectations from surgical interventions [25]. These individuals do not consider walking as a treatment—this is an illustration of the limited education about their condition that they are receiving from health care professionals [25]. Patients often feel the need to seek further information from friends, family, and the internet, making it even more important for web-based information to be accurate and comprehensive [26].

This perhaps also highlights a wider issue related to education on and the management of PAD and IC. Health care professionals, often in the context of limited resources, may refer their patients to web-based information, who in turn believe that the sources are comprehensive and accurate. This may serve as a substitute for or to supplement the education provided in the clinic. The findings of this review indicate that even websites normally considered reliable—for example, those of the AHA, British Heart Foundation, and Mayo clinic (see Multimedia Appendices 1-3)—have substantial limitations. Further engagement of specialized clinicians and educators in developing, reviewing, and signposting educational resources is required and may contribute to improved knowledge even among health care professionals [27].

After smoking cessation, exercise is arguably the next most important recommended self-management treatment for PAD and IC [28]. Therefore, it is promising that many websites (51/62, 82%) recommended this accurately. The literature suggests that supervised exercise programs (SEPs) are more beneficial to people with PAD and those with IC than general advice on home exercise [29,30]. However, SEPs have been much less frequently and appropriately endorsed by websites (21/62, 34%). Even though SEPs are endorsed by the NICE and AHA guidelines [3,16], lack of resources and funding often prevent their widespread use in practice [31]. Therefore, recommendations on websites alone are not enough to improve the management of PAD. There needs to be cohesion among guidelines, website recommendations, and the availability of health care resources to allow the provision of optimal care for patients with PAD.

Worldwide, the HON Foundation is recognized as an organization that assesses the quality of web-based health information directed at patients. In this study, only a small proportion of websites presenting PAD and IC treatment recommendations displayed the HONcode certificate logo (8%), which is lower than that reported for websites providing information regarding idiopathic pulmonary fibrosis (15%) and low back pain (41%) [8,11]. Importantly, HON Foundation–certified websites were not drastically better or more or less readable than noncertified websites. They tended to include more than the average number of accurate recommendations (8/21) but still also included many unclear recommendations. This suggests that the HONcode does not completely reflect the quality or accuracy of websites providing health information, which adds to the challenge of determining the accuracy of web-based health information for patients.

A significant concern regarding web-based health information is how accessible this information is to the average reader. Previous research has found most health websites do not have acceptable readability levels, including those designed for people with PAD [12]. Studies assessing web-based information on inflammatory bowel disease and pancreatic cancer found that only 4%-5% of websites were “readable,” as revealed by a FKGL of <8 [9,18]. Similarly, only 5% of websites in this review achieved this acceptable reading level. The average FKGL of websites supplying PAD and IC health information was 11.2, which is much higher than the grade 6-8 level recommended for this type of information. Increased accuracy of websites is associated with increased reading level scores, and it seems to be difficult to produce accurate and easily understood information for all audiences [32]; however, it is important that all those who provide medical information via web-based resources are aware of the importance of providing both accurate and readable content.

### Limitations

In this study, the only search engine used was Google as it is known to have the best search validity and is the most popular search engine [10,15]. Multiple search engines have been used in other studies to enhance the likelihood of finding all relevant websites. The literature on this is conflicting; however, studies have shown that only 1% of first-page results were the same when searched on both Google and Yahoo [18], with a high degree of overlap between results from different search engines [10]. Furthermore, the findings of this review may be limited from a global perspective owing to only seeking websites presented in English and only from US- and UK-specific website domains. However, as we were comparing website recommendations to the NICE and AHA guidelines, it was appropriate to use websites from corresponding countries. Using specific search terms to target “trustworthy” websites could be a limitation as the average person searching for this information would be unlikely to use these specific search terms. However, this meant that the recommendations by the websites are more likely to be trusted and followed by individuals. In this study, ranking of search results and website layout and design were not evaluated, but it is likely that this may also affect a person's ability to access accurate information.

### Conclusions

Websites recommending treatments and management of PAD and IC are mostly accurate but have low credibility, low comprehensiveness, and are too complex for the average person to understand. With an increasing number of individuals seeking health information on the internet, it is imperative that websites be of high quality and do not act as barriers to patient education or introduce bias or unrealistic expectations for care. Rather, they should support self-management and behavior change and should reflect the advice and treatment options provided by health care professionals. Websites presenting information on PAD and IC should do so in accordance with evidence-based guidelines as much as possible, and health care professionals must ensure that they are providing clear and complete information to people with PAD and IC to avoid them from lacking an understanding of their condition. Future research should further assess available web-based information on PAD and IC, as well as overall patient and professional perceptions of the condition.

## Appendix

Multimedia Appendix 1 Credibility of websites as measured by the JAMA benchmark.

Multimedia Appendix 2 Frequency (%) of websites endorsing or dismissing treatments mentioned in NICE or AHA guidelines (n=62).

Multimedia Appendix 3 Accuracy and Comprehensiveness of website recommendations.

## Abbreviations

AHA: American Heart Association
FKGL: Flesch–Kincaid grade level
HON: Health On the Net
HONcode: Health On the Net Foundation’s code of conduct
IC: intermittent claudication
JAMA: Journal of the American Medical Association
NICE: National Institute for Health and Care Excellence
PAD: peripheral artery disease
SEP: supervised exercise program
