# Supplementary material for: Credibility, Accuracy, and Comprehensiveness of Readily Available Internet-Based Information on Treatment and Management of Peripheral Artery Disease and Intermittent Claudication: Review
Source: J Med Internet Res. 2022 Oct 17;24(10):e39555. doi: 10.2196/39555 (PMC9623463; doi:10.2196/39555)
Supplement: Multimedia Appendix 1 [file jmir_v24i10e39555_app1.docx]

**Multimedia Appendix 1. Credibility of websites as measured by the JAMA benchmark. Y - Yes, N - No, NR - Not reported. JAMA: Journal of the American Medical Association.**

| **JAMA Benchmark** | **Updated after NICE guidelines** | **Authorship Declared** | **Reference List** | **Disclosure of Interest/Funding** |
| --- | --- | --- | --- | --- |
| Peripheral Artery Disease UK |  |  |  |  |
| NHS | Y | N | N | N |
| BUPA | NR | Y | N | N |
| British Heart Foundation (BHF) | NR | N | N | N |
| Patient Platform ltd | Y | Y | Y | N |
| Circulation Foundation | NR | N | N | N |
| Guidelines | Y | Y | N | Y |
| British Heart Foundation (BHF)/NHS | NR | N | N | N |
| University of Kentucky Healthcare | NR | N | N | N |
| Patient Platform ltd | Y | Y | Y | N |
| The Vascular Society for Great Britain and Ireland | NR | N | N | N |
| Society for Vascular Surgery | NR | Y | N | N |
| Healthline | Y | Y | Y | N |
| Northern Care alliance NHS | Y | N | N | N |
| Top Doctors | Y | Y | N | N |
| Vascular News | Y | N | N | N |
| Peripheral Artery Disease USA |  |  |  |  |
| Centres for Disease Control and Prevention (CDC) | NR | N | Y | N |
| National Heart, Lung and Blood Institute (NHLBI) | Y | N | Y | N |
| Winchester Hospital | NR | N | Y | N |
| Victoria State Government | Y | N | Y | N |
| American Heart Association | Y | N | N | N |
| Mayo Clinic | Y | N | Y | N |
| Cleveland Clinic | Y | N | N | N |
| Stanford Healthcare | NR | N | N | N |
| John Hopkins Medicine | NR | N | N | N |
| The Carle Foundation | NR | N | N | N |
| University Hospitals | NR | N | N | N |
| Brigham and Women’s Hospital | NR | N | N | N |
| Massachusetts General Hospital | NR | N | N | N |
| Mount Sinai Hospitals | NR | N | N | N |
| Dignity Health | NR | N | N | N |
| Barnes Jewish Hospital | NR | N | N | N |
| Duke Health | Y | N | N | N |
| University of Chicago | NR | N | N | N |
| University of Missouri Healthcare | NR | N | N | N |
| MedStar Health | NR | N | N | N |
| University of Miami Health System | NR | N | N | N |
| MSD manuals | Y | Y | N | N |
| Memorial Hermann Heart and Vascular Institute | NR | N | N | N |
| Harvard Medical School | N | N | N | N |
| Medical News Today | Y | Y | Y | N |
| Radiology Info | Y | Y | N | N |
| University of Kansas health System | N | N | N | N |
| Medtronic | Y | N | N | N |
| Intermittent Claudication UK |  |  |  |  |
| Circulation Foundation | NR | N | N | N |
| NI direct | NR | N | N | N |
| Leeds Teaching Hospital NHS | NR | N | N | N |
| Sandwell and West Birmingham Hospitals NHS | Y | N | Y | N |
| Royal Berkshire NHS | Y | Y | N | N |
| North Bristol NHS | Y | Y | N | N |
| Dudley Group NHS | N | N | N | N |
| Veins Wales | N | N | N | N |
| Norfolk and Norwich University Hospitals | Y | Y | N | N |
| Coventry and Warwickshire NHS | Y | Y | N | N |
| NHS Wales | Y | N | N | N |
| Intermittent Claudication USA |  |  |  |  |
| Mount Sinai Hospitals | NR | N | Y | N |
| Up to Date | Y | Y | Y | N |
| Healthline | Y | Y | Y | N |
| St Luke's Hospital | NR | N | Y | N |
| Westchester Medical Centre | NR | N | N | N |
| University of Nebraska Medical Centre | NR | N | N | N |
| University of California (San Francisco) | N | Y | N | N |
| Government of Alberta | Y | Y | Y | N |
