# Supplementary material for: Credibility, Accuracy, and Comprehensiveness of Readily Available Internet-Based Information on Treatment and Management of Peripheral Artery Disease and Intermittent Claudication: Review
Source: J Med Internet Res. 2022 Oct 17;24(10):e39555. doi: 10.2196/39555 (PMC9623463; doi:10.2196/39555)
Supplement: Multimedia Appendix 2 [file jmir_v24i10e39555_app2.docx]

**Multimedia Appendix 2. Frequency (%) of websites endorsing or dismissing treatments mentioned in NICE or AHA guidelines (n=62). AHA: American Heart Association. NICE: National Institute for Health and Care Excellence.**

| Treatments | Appropriate Endorsements n(%) | | | Appropriate Dismissals  n(%) | | Inappropriate Endorsements n(%) | | Inappropriate Dismissals  n(%) | Unclear  n(%) | | | Omissions  n(%) |
| --- | --- | --- | --- | --- | --- | --- | --- | --- | --- | --- | --- | --- |
| Treatments endorsed by at least 1 guideline | | |  | |  | |  | | |  |  |  |
| Physical activity/exercise | | 51 (82.3) | | - | | - | | - | 1 (1.6) | | 10 (16.7) | |
| Supervised exercise program | | 21 (33.9) | | - | | - | | - | 2 (3.2) | | 38 (61.3) | |
| Exercise to maximal pain | | 6 (9.7) | | - | | - | | - | 2 (3.2) | | 54 (87.1) | |
| Diabetes Management | | 36 (58.1) | | - | | - | | - | - | | 26 (41.9) | |
| Antiplatelet Therapy | | 41 (66.1) | | - | | - | | - | 1 (1.6) | | 20 (32.3) | |
| Angioplasty | | 52 (83.9) | | - | | - | | - | - | | 10 (16.7) | |
| Bypass surgery | | 49 (79) | | - | | - | | - | 1 (1.6) | | 12 (19.4) | |
| Stent placement for IC caused by aorto-iliac occlusion | | - | | - | | - | | - | 32 (51.6) | | 30 (48.4) | |
| Vasodilators | | 26 (41.9) | | - | | - | | - | - | | 36 (58.1) | |
| Stop smoking | | 53 (85.5) | | - | | - | | - | - | | 9 (14.5) | |
| Cholesterol Management | | 53 (85.5) | | - | | - | | - | - | | 9 (14.5) | |
| BP Management | | 51 (82.3) | | - | | - | | - | - | | 11 (17.7) | |
| Healthy Diet | | 36 (58.1) | | - | | - | | - | 1 (1.6) | | 25 (40.3) | |
| Weight Management | | 29 (46.8) | | - | | - | | - | - | | 33 (53.2) | |
| Annual Flu Vaccine | | - | | - | | - | | - | - | | 62 (100) | |
| Foot Care | | 10 (16.7) | | - | | - | | - | - | | 52 (83.9) | |
| Treatments dismissed by at least 1 guideline | | | | | | | | | | | | |
| B Complex Vitamins | | - | | 5 (8.1) | | - | | - | - | | 57 (91.9) | |
| Pentoxifylline | | - | | 2 (3.2) | | 5 (8.1) | | - | 3 (4.8) | | 52 (83.9) | |
| Chelation Therapy | | - | | 2 (3.2) | | - | | - | - | | 60 (96.8) | |
| Anti-Coagulants | | - | | 3 (4.8) | | 2 (3.2) | | - | 2 (2.9) | | 55 (88.7) | |
| Stenting for IC caused by aorto-iliac disease or femoro-popliteal disease | | - | | - | | - | | - | 32 (51.6) | | 30 (48.4) | |
