# Supplementary material for: Credibility, Accuracy, and Comprehensiveness of Readily Available Internet-Based Information on Treatment and Management of Peripheral Artery Disease and Intermittent Claudication: Review
Source: J Med Internet Res. 2022 Oct 17;24(10):e39555. doi: 10.2196/39555 (PMC9623463; doi:10.2196/39555)
Supplement: Multimedia Appendix 3 [file jmir_v24i10e39555_app3.docx]

**Multimedia Appendix 3. Accuracy and Comprehensiveness of website recommendations. AE – appropriate endorsement, AD – Appropriate Dismissal, IE – inappropriate Endorsement, ID – Inappropriate Dismissal, UC – Unclear, E – Endorsement, D – Dismissal.**

| Name of website | Number of Recommendations given by website | AE | AD | IE | ID | UC | E | D | Number of clear and accurate recommendations | Number of recommendations from guidelines correctly covered by website n(%) |
| --- | --- | --- | --- | --- | --- | --- | --- | --- | --- | --- |
| NI direct | 10 | 8 | 0 | 0 | 0 | 2 | 1 | 0 | 8 | 8 (38.1) |
| NHS | 15 | 13 | 0 | 0 | 0 | 0 | 2 | 0 | 13 | 13 (61.9) |
| BUPA | 13 | 12 | 0 | 0 | 0 | 3 | 0 | 0 | 12 | 12 (57.1) |
| British Heart Foundation (BHF) | 10 | 7 | 0 | 0 | 0 | 2 | 1 | 0 | 7 | 7 (33.3) |
| Patient Platform ltd | 13 | 12 | 0 | 0 | 0 | 0 | 1 | 0 | 12 | 12 (57.1) |
| Circulation Foundation | 8 | 7 | 0 | 0 | 0 | 2 | 0 | 0 | 7 | 7 (33.3) |
| Guidelines | 12 | 9 | 0 | 0 | 0 | 1 | 1 | 1 | 10 | 9 (42.9) |
| British Heart Foundation (BHF)/NHS | 17 | 13 | 0 | 0 | 0 | 3 | 2 | 0 | 13 | 13 (61.9) |
| University of Kentucky Healthcare | 8 | 7 | 0 | 0 | 0 | 0 | 2 | 0 | 7 | 7 (33.3) |
| Patient Platform ltd | 17 | 11 | 2 | 0 | 0 | 2 | 1 | 2 | 15 | 13 (61.9) |
| The Vascular Society for Great Britain and Ireland | 11 | 9 | 0 | 0 | 0 | 3 | 0 | 0 | 9 | 9 (42.9) |
| Society for Vascular Surgery | 10 | 9 | 0 | 0 | 0 | 2 | 0 | 0 | 9 | 9 (42.9) |
| Healthline | 11 | 10 | 0 | 1 | 0 | 3 | 0 | 0 | 10 | 10 (47.6) |
| Northern Care alliance NHS | 11 | 10 | 0 | 0 | 0 | 1 | 0 | 0 | 10 | 10 (47.6) |
| Top Doctors | 7 | 7 | 0 | 0 | 0 | 0 | 0 | 0 | 7 | 7 (33.3) |
| Vascular News | 5 | 5 | 0 | 0 | 0 | 0 | 0 | 0 | 5 | 5 (23.8) |
| Centres for Disease Control and Prevention (CDC) | 8 | 8 | 0 | 0 | 0 | 0 | 0 | 0 | 8 | 8 (38.1) |
| National Heart, Lung and Blood Institute (NHLBI) | 17 | 13 | 0 | 1 | 0 | 2 | 2 | 0 | 13 | 13 (61.9) |
| Winchester Hospital | 19 | 9 | 0 | 2 | 0 | 2 | 2 | 2 | 11 | 9 (42.9) |
| Victoria State Government | 15 | 10 | 0 | 1 | 0 | 3 | 2 | 0 | 10 | 10 (47.6) |
| American Heart Association | 13 | 9 | 0 | 0 | 0 | 5 | 1 | 0 | 9 | 9 (42.9) |
| Mayo Clinic | 16 | 12 | 0 | 1 | 0 | 2 | 1 | 0 | 12 | 12 (57.1) |
| Cleveland Clinic | 15 | 13 | 0 | 0 | 0 | 2 | 1 | 0 | 13 | 13 (61.9) |
| Stanford Healthcare | 16 | 10 | 0 | 0 | 0 | 2 | 5 | 0 | 10 | 10 (47.6) |
| John Hopkins Medicine | 12 | 10 | 0 | 0 | 0 | 2 | 1 | 0 | 10 | 10 (47.6) |
| The Carle Foundation | 13 | 11 | 0 | 0 | 0 | 3 | 0 | 0 | 11 | 11 (52.4) |
| University Hospitals | 10 | 8 | 0 | 0 | 0 | 2 | 1 | 0 | 8 | 8 (38.1) |
| Brigham and Womens Hospital | 11 | 9 | 0 | 0 | 0 | 2 | 0 | 0 | 9 | 9 (42.9) |
| Massachussetts General Hospital | 10 | 7 | 0 | 0 | 0 | 3 | 1 | 0 | 7 | 7 (33.3) |
| Mount Sinai Hospitals | 8 | 5 | 0 | 0 | 0 | 2 | 2 | 0 | 5 | 5 (23.8) |
| Dignity Health | 13 | 10 | 0 | 0 | 0 | 1 | 2 | 0 | 10 | 10 (47.6) |
| Barnes Jewish Hospital | 6 | 3 | 0 | 0 | 0 | 2 | 0 | 0 | 3 | 3 (14.3) |
| Duke Health | 11 | 7 | 0 | 0 | 0 | 2 | 1 | 0 | 7 | 7 (33.3) |
| University of Chicago Medical Centre | 3 | 2 | 0 | 0 | 0 | 1 | 0 | 0 | 2 | 2 (9.5) |
| University of Missouri Healthcare | 8 | 7 | 0 | 0 | 0 | 2 | 0 | 0 | 6 | 6 (28.6) |
| MedStar Health | 6 | 3 | 0 | 0 | 0 | 1 | 2 | 0 | 3 | 3 (14.3) |
| University of Miami Health Syste, | 7 | 6 | 0 | 0 | 0 | 2 | 0 | 0 | 6 | 6 (28.6) |
| MSD manuals | 10 | 6 | 0 | 0 | 0 | 4 | 1 | 2 | 8 | 8 (38.1) |
| Memorial Hermann Heart and Vascular Institute | 8 | 6 | 0 | 0 | 0 | 2 | 1 | 0 | 6 | 6 (28.6) |
| Harvard Medical School | 13 | 10 | 0 | 1 | 0 | 2 | 0 | 1 | 11 | 10 (47.6) |
| Medtronic | 12 | 10 | 0 | 0 | 0 | 3 | 1 | 0 | 10 | 10 (47.6) |
| Medical News Today | 8 | 8 | 0 | 1 | 0 | 0 | 0 | 0 | 8 | 8 (38.1) |
| Radiology Info | 8 | 5 | 0 | 0 | 0 | 2 | 2 | 0 | 5 | 5 (23.8) |
| University of Kansas health System | 6 | 2 | 0 | 0 | 0 | 2 | 3 | 0 | 2 | 2 (9.5) |
| Circulation Foundation | 7 | 6 | 0 | 0 | 0 | 1 | 0 | 0 | 6 | 6 (28.6) |
| Leeds Teaching Hospital NHS | 6 | 5 | 0 | 1 | 0 | 0 | 0 | 0 | 5 | 5 (23.8) |
| Sandwell and West Birmingham Hospitals NHS | 8 | 8 | 0 | 0 | 0 | 0 | 0 | 0 | 8 | 8 (38.1) |
| Royal Berkshire NHS | 8 | 7 | 0 | 0 | 0 | 1 | 0 | 0 | 7 | 7 (33.3) |
| North Bristol NHS | 13 | 10 | 0 | 0 | 0 | 3 | 0 | 0 | 10 | 10 (47.6) |
| Veins Wales | 9 | 9 | 0 | 0 | 0 | 1 | 0 | 0 | 9 | 9 (42.9) |
| Dudley Group NHS | 7 | 7 | 0 | 0 | 0 | 0 | 0 | 0 | 7 | 7 |
| NHS Wales | 8 | 8 | 0 | 0 | 0 | 0 | 0 | 0 | 8 | 8 (38.1) |
| Coventry and Warwickshire NHS | 10 | 10 | 0 | 0 | 0 | 0 | 0 | 0 | 10 | 10 (47.6) |
| Norfolk and Norwich University Hospitals NHS | 8 | 7 | 0 | 0 | 0 | 1 | 0 | 0 | 7 | 7 (33.3) |
| Mount Sinai Hospitals | 15 | 10 | 2 | 0 | 0 | 3 | 0 | 3 | 15 | 12 (57.1) |
| Up to Date | 17 | 10 | 2 | 0 | 0 | 4 | 0 | 2 | 14 | 12 (57.1) |
| Healthline | 9 | 8 | 0 | 0 | 0 | 2 | 0 | 0 | 8 | 8 (38.1) |
| St Luke's Hospital | 17 | 10 | 2 | 0 | 0 | 4 | 0 | 2 | 14 | 12 (57.1) |
| Westchester Medical Centre | 9 | 5 | 0 | 0 | 0 | 4 | 0 | 0 | 5 | 5 (23.8) |
| University of California (San Francisco) | 11 | 10 | 0 | 0 | 0 | 2 | 0 | 0 | 10 | 10 (47.6) |
| University of Nebraska Medical Centre | 13 | 10 | 0 | 1 | 0 | 3 | 1 | 0 | 10 | 10 (47.6) |
| Government of Alberta | 12 | 11 | 0 | 0 | 0 | 1 | 0 | 0 | 11 | 11 (52.4) |
